# Progress in Medicine

**Published:** 1900-10-06

**Authors:** 


### Progress in Medicine.

### DISEASES OF THE RESPIRATORY SYSTEM.

**Pneumonia.**—One out of every 18 deaths from all causes is registered as due to pneumonia.[1] The incidence and mortality of the disease are highest during the first year of life, and again become heavy after the age of 55. Males suffer more than females, and natives of the tropics more than Europeans. Exposure to dusty atmospheres and alcoholism produce a high death rate, and the disease is more rife in cold than in hot weather. Pneumonia is sometimes infectious and takes on epidemic form.

That the disease is constantly associated with the presence of the pneumococcus of Fraenkel is well established, but whether this be the true causative agent is uncertain. It is not necessary that the organism should enter by the respiratory tract, for the disease is a blood infection, the organisms finding in the lungs the most suitable site for multiplication. This has been demonstrated by Schultz,[2] who inoculated 14 animals directly through the jugular vein, pneumia supervening in eight, although there was no antecedent alteration brought about in the lungs by chill or injury. With regard to the symptomology of this disease there is little fresh to be said: both Broadbent and Barr[3] have, however, recently pointed out that in apical pneumonia the appearance of physical signs takes place very late, but supervenes then in a most marked degree and with great rapidity. The crisis does not coincide with the improvement in the lung. In children and in adults, even before the stage of consolidation, increased resistance on the affected side may be made out by palpation. In order to arrest all vibration of the chest walls, detect the slightest increase of resistance and elicit the impaired note of the congested lung underneath, Barr suggests than in examining children the whole of the left hand be placed on the chest and then the middle finger percussed.

From an analysis of 750 cases Sello[4] finds that the commonest complication of pneumonia is pleurisy. This was serious in 8·7 per cent. of the cases and purulent in 4·5 per cent., the fluid as a rule in the former condition being sterile. The cases of endocarditis and meningitis were fatal, so also were six cases of nephritis, in all of which the pneumococcus was present. Of 11 cases of abscess of the lung seven recovered. Two out of three cases of gangrene of the lung succumbed. Three patients developed hemiplegia, in two the cause being probably an embolus, whilst the third resembled thrombosis. An unusual complication of pneumonia is mentioned by Berthier,[5] viz., gangrene of the fingers and toes. This may supervene between the third and tenth day after the crisis. Its onset is extremely sudden, and very little pain is felt. The gangrene is generally of the dry form, a line of demarcation appears, and the digits separate spontaneously. Gangrene of the lung follows pneumonia in about 3 per cent. of fatal cases. Levings[6] states that there are no pathognomonic symptoms until the gangrenous area has opened into the bronchus. There is then hæmorrhage, fœtor of the breath, and a sweetish disagreeable taste in the patient's mouth. The sputum on standing consists of three layers, the upper mucous, the second serous, and the lowest loaded with débris. There are now signs of a consolidated patch in the lung going on to cavitation. The fluid in the cavity shifts with each alteration in position, so that, according to Wintrich, if the mouth be open a rise in pitch is noticed upon percussion only in certain positions, as, for instance, lying upon one side or sitting up. 30 per cent. of cases recover without operation, and 70 to 75 per cent. of those operated upon recover. The indications for operations are: failure to drain through a bronchus, severe hæmorrhage, exhaustion of the patient, harassing cough, irritative diarrhœa, and commencing hectic. With regard to prognosis, Barr, to whose lectures we have already referred, states that the mortality from pneumonia is from 15–25 per cent. Over 60 years of age it is 50 per cent. Prognosis, according to Broadbent,[7] is bad in old age, debility, obesity, alcoholism, diabetes, renal or cardiac disease. A distended abdomen from any cause is unfavourable. Delirium is always serious, and is more frequent in apical cases. A sudden onset with herpes labialis is generally favourable.

Gairdner[8] relies chiefly on expectant treatment in pneumonia, using mild expectorants and diuretics. Opium is contraindicated, in his opinion. Barr, on the other hand, believes that nothing but good can accrue from a free administration of opium early in the disease. For the reduction of temperature he recommends cold sponging and the application of large ice bags to the abdomen, the groins first being protected by cotton wool. In the first three days antimony has a powerful effect in checking the inflammation. Ten minims of antimonial wine may be given every 4 hours to an adult. Strychnine, caffeine and digitalis are employed for heart failure, but alcohol is better withheld. Free ventilation of the sick room is essential. The antiseptic treatment of pneumonia is favoured by Smith.[9] Amongst the drugs used are small repeated doses of calomel, salicylates, quinine, inhalations of chloroform and creosote or some of its modifications. Antipneumococcus serum is so difficult to obtain that reports on this system of medication are very scanty. Fanoni[10] reports a series of cases all treated with serum. The results were uniformly satisfactory, the general condition being improved and resolution hastened.

**Treatment of Asthma.**—Noorden[11] recommends the use of atropine in this disease. The treatment is commenced with ½-minim doses of liquor atropinæ sulphatis, and the dose increased every second or third day until a maximum of 6 minims is reached. The quantity is then gradually reduced, and treatment stopped, to be resumed in six months' time. Patients under treatment require to be closely watched, but as a rule no bad effects result from the exhibition of the drug.

Laudi[12] recognises a form of asthma in neurotic children due to dyspepsia. In such cases there is an increased bulbar receptivity and reflex excitability. The tongue is furred, breath foul, and nausea, vomiting and acetonuria are present. Speedy relief is afforded by the exhibition of purgatives and emetics.

Paraldehyde relieves the spasm of asthma and induces sleep without objectionable after effects. Macgregor[13] recommends that, whilst this is given at night, iodide of potash combined with tincture of lobelia be taken during the day. The nauseous taste of the drug may be disguised by common water and tincture of orange peel. Wynter[14] suggests equal parts of paraldehyde and expressed oil of almond. It may also be given in milk, beer or wine. The dose recommended is 1 drachm.

Where asthma is dependent upon bronchitis and the presence of mucus, Jackson[15] advises the use of potassium iodide in 10-grain doses every two hours during the attacks and of ichthyol between the attacks. During the spasm $\frac{1}{8}$ gr. of morphine sulphate combined with $\frac{1}{150}$ gr. atropine sulphate, repeated in one-half to one, may give relief. Bromides, chloral and bromoform also give good results, as do the burning of medicated powders. Where the asthma is a reflex from coryza or hay-fever the nasal cavities should be syringed with Dobell's solution or Listerine, and sprayed with a 4 per cent. solution of cocaine.

**Bronchitis.**—Closely allied to asthma in the spasmodic nature of the attacks is a catarrhal inflammation of the bronchi described by Teichmüller[16] as eosinophile bronchitis. This form of disorder is found chiefly amongst strumous, syphilitic or alcoholic subjects. There is slight elevation of temperature, headache, anorexia, vomiting, constipation and sweating. Pain in the chest is a common symptom, and on auscultation there may be heard moist rales, or râles, snoring and wheezing in character. The distinctive feature of the disease is the sputum. This is transparent, viscid and loose, and crowded with eosinophile cells, a condition met with in no other pulmonary state. The disease is chronic in its course, but generally improves under hygienic and dietetic treatment. Schmidt[17] reports a case of chronic fibrinous bronchitis which followed a chill and severe fright. The patient also suffered from gravel and eczema. A large number of fibrinous branched plugs were coughed up daily. These cylindrical plugs contained erythrocytes, leucocytes and mucous corpuscles. They also contained crystals of cholesterin and small shining bodies which were named corpora lecithinœdea. These last-named bodies were soluble in ether, chloroform and carbon disulphide.

An unusual complication of bronchitis, namely, subcutaneous emphysema, is described by Gordon.[18] The patient was a child four and a-half years of age, and on the third day of an attack of bronchitis a swelling was noticed on the right side of the neck. This proved to be subcutaneous emphysema, and rapidly spread over the whole face and chest. There had been no severe paroxysms of coughing to account for the lesion. At first there was high temperature, dyspnœa, rapid pulse and jactitation, but as the swelling diminished these symptoms subsided, and by the tenth day the child was convalescent. Treatment consisted in 2 gr. doses of antifebrin every four hours until the temperature was normal. For the first three days poultices were applied to the chest; subsequently it was rubbed morning and evening with neatsfoot oil.

[1] Practitioner, June, 1899. [2] Arch. des Sc. Biolog., T. viii., No. 1. [3] Brit. Med. Jour., June 9, 1900. [4] Zeit. K. F. Med., xxxvi. [5] Thèse de Paris, 1899. [6] N. Y. Med. Jour., Oct. 14, 1899. [7] Practitioner, June, 1899. [8] Ibid. [9] Med. News, Dec. 16, 1899. [10] N. Y. Med. Jour., Aug. 26, 1899. [11] Therap. Monatsh., Oct., 1898. [12] Clin. Mod. An. 5, N. 24. [13] N. Y. Lancet, 1899, page 127. [14] Treatment, Sept. 14, 1899. [15] Merck's Arch., Dec., 1899. [16] Deut. Arch. f. Kl. Med. B. 63, H. 5. [17] Centrab. Path. Bx. Nos. 11 and 12. [18] Lancet, Dec. 9, 1899.

